# Campylobacteriosis in Urban versus Rural Areas: A Case-Case Study Integrated with Molecular Typing to Validate Risk Factors and to Attribute Sources of Infection

**DOI:** 10.1371/journal.pone.0083731

**Published:** 2013-12-26

**Authors:** Simon Lévesque, Eric Fournier, Nathalie Carrier, Eric Frost, Robert D. Arbeit, Sophie Michaud

**Affiliations:** 1 Department of Microbiology and Infectious Diseases, Faculté de Médecine de l'Université de Sherbrooke, Québec, Canada; 2 Laboratoire de santé publique du Québec, Institut national de santé publique du Québec, Sainte-Anne-de-Bellevue, Québec, Canada; 3 Centre de Recherche Clinique Étienne-Le Bel du Centre Hospitalier Universitaire de Sherbrooke, Sherbrooke, Québec, Canada; 4 Infectious Diseases Section, Tufts University School of Medicine, Boston, Massachusetts, United States of America; Cornell University, United States of America

## Abstract

*Campylobacter* infection is a leading cause of bacterial gastroenteritis worldwide, and most clinical cases appear as isolated, sporadic infections for which the source is rarely apparent. From July 2005 to December 2007 we conducted a prospective case-case study of sporadic, domestically-acquired *Campylobacter* enteritis in rural versus urban areas and a prevalence study of *Campylobacter* in animal and environmental sources in the Eastern Townships, Quebec. Isolates were typed using Multilocus Sequence Typing (MLST) to reinforce the case-case findings and to assign a source probability estimate for each human isolate. The risk of human campylobacteriosis was 1.89-fold higher in rural than urban areas. Unconditional multivariate logistic regression analysis identified two independent risk factors associated with human *Campylobacter* infections acquired in rural area: occupational exposure to animals (OR = 10.6, 95% CI: 1.2–91, *p* = 0.032), and household water coming from a private well (OR = 8.3, 95% CI: 3.4–20.4, *p*<0.0001). A total of 851 *C. jejuni* isolates (178 human, 257 chicken, 87 bovine, 266 water, 63 wild bird) were typed using MLST. Among human isolates, the incidence rates of clonal complexes (CC) CC-21, CC-45, and CC-61 were higher in rural than urban areas. MLST-based source attribution analysis indicated that 64.5% of human *C. jejuni* isolates were attributable to chicken, followed by cattle (25.8%), water (7.4%), and wild birds (2.3%). Chicken was the attributable source for the majority of cases, independent of residential area, sex and age. The increased incidence in rural compared to urban areas was associated with occupational exposure to animals, particularly cattle among those aged 15–34 years, and with consumption of private well water. Both bovine and water exposure appeared to contribute to the seasonal variation in campylobacteriosis. These results provide a basis for developing public education and preventive programs targeting the risk factors identified.

## Introduction

*Campylobacter* infection is a leading cause of bacterial gastroenteritis worldwide. Canada has reported an average of 39 cases per 100,000 inhabitants annually in the last decade [1] and United States reports 13,000 hospitalizations and over 100 deaths each year [2]. *Campylobacter* colonizes the digestive tract of a wide range of warm-blooded animal hosts, including all major domestic animals and wildlife, and the feces of infected animals are responsible for the greatest environmental burden of campylobacters [3]. Poultry, raw milk and untreated water are well-documented sources of human campylobacteriosis outbreaks [4]. However, most cases appear as isolated, sporadic infections for which the source is rarely apparent. Identifying the sources and routes of transmission of campylobacteriosis is essential for developing effective, targeted preventive measures.

Clinical descriptive data are frequently insufficient to identify sources of sporadic campylobacteriosis, at least in part, because of the delay between the onset of symptoms and the epidemiological investigation [5]–[7]. Molecular strain typing provides a complementary approach for studying the epidemiology of campylobacteriosis and for defining the likely sources of infection [8], [9]. Multi-locus sequence typing (MLST) is a robust genotyping method that can be used to identify connections in the core-genome of *Campylobacter* isolates from apparently unrelated, sporadic cases encountered in routine surveillance [8]–[10]. Some *Campylobacter* sequence types (STs) are strongly associated with a particular reservoir. For example, ST-61 has been found to be associated with cattle, ST-257 with chicken, ST-177 with wild birds, and ST-3704 with bank voles [8], [11]–[14]. Such associations allow to use MLST to quantitatively estimate the likely sources of human *Campylobacter* infection [4], [15], [16].

Studies in multiple countries have documented that human *Campylobacter* infections are appreciably more common in rural rather than urban areas, initially with clinical descriptive data [9], [17]–[20], and then with molecular strain typing (21–23). In Ontario in 1978 through 1985, Thompson et al. [17] reported rates of 350-400/100,000 in some rural areas compared to 80 cases/100,000 inhabitants in urban ones and identified raw milk consumption as an important risk factor. In Manitoba in 1996 through 2004, Green et al. [21] reported 44 cases/100,000 inhabitants in rural areas compared to 14 cases/100,000 inhabitants in urban ones and suggested that raw milk and well water consumption might be the causes.

Molecular strain typing has confirmed that chicken consumption is the main risk factor for human *Campylobacter* infection. When demographic data were considered, only children showed an urban-rural gradient, where chicken was a major source of infection in young urban children, but not in their rural counterparts, for which ruminants and other avian sources were more important [16], [22], [23]. It was also suggested that consumption of chicken, rather than contact with live animals, was the main risk factor.

Here we conducted a prospective case-case study of domestically-acquired *Campylobacter* enteritis in the Eastern Townships, Quebec, to test the hypothesis that some risk factors – such as handling raw chicken or eating undercooked poultry – are common exposures to both urban and rural areas, whereas other risk factors are responsible for the excess of human cases in rural areas. In parallel, we conducted a prevalence study of *Campylobacter* in animal and environmental sources of human infection (retail fresh whole chicken, environmental water, wild birds, and cattle). Finally, we typed a number of *Campylobacter* isolates from human cases, animal and environmental sources using MLST as to reinforce the findings of the case-case study and to determine the likely sources of sporadic human *Campylobacter* infections in the Eastern Townships, Quebec.

## Results

### Human campylobacteriosis cases

From July 2005 to December 2007, 352 human campyloabacteriosis cases were reported. Of these, 111 were excluded: 41 cases acquired their infection outside Quebec, 19 resided outside the Eastern Townships, 49 could not be interviewed or declined to participate, and two were cases with reinfections. Consequently, 350 cases were used to calculate the crude incidence rate and 241 cases of campylobacteriosis were included in the case-case comparison.

Table 1 shows the *Campylobacter* species distribution of human isolates. Overall, 219 (90.9%) of the isolates were identified as *C. jejuni*. The two *C. fetus* isolates were obtained from blood cultures; all the remaining isolates were cultured from stool. Clinical presentation of cases is shown in supplemental material.

**Table 1:** *Campylobacter* isolates obtained from human cases and from the sampled animal reservoirs.

Species	Human	Chicken	Bovine	Wild bird
	(N = 241)	(N = 371)	(N = 174)	(N = 108)
*C. jejuni*	219 (91%)	332 (89%)	126 (72%)	78 (72%)
*C. coli*	11 (5%)	37 (10%)	5 (3%)	9 (8%)
*C. lari*	1 (<1%)	-	6 (4%)	14 (13%)
*C. fetus*	2 (1%)	-	35 (20%)	1 (1%)
*C. upsaliensis*	3 (1%)	-	-	1 (1%)
*C.* sp.	5 (2%)	2 (1%)	2 (1%)	5 (5%)

**Figure 1.Quarterly:**
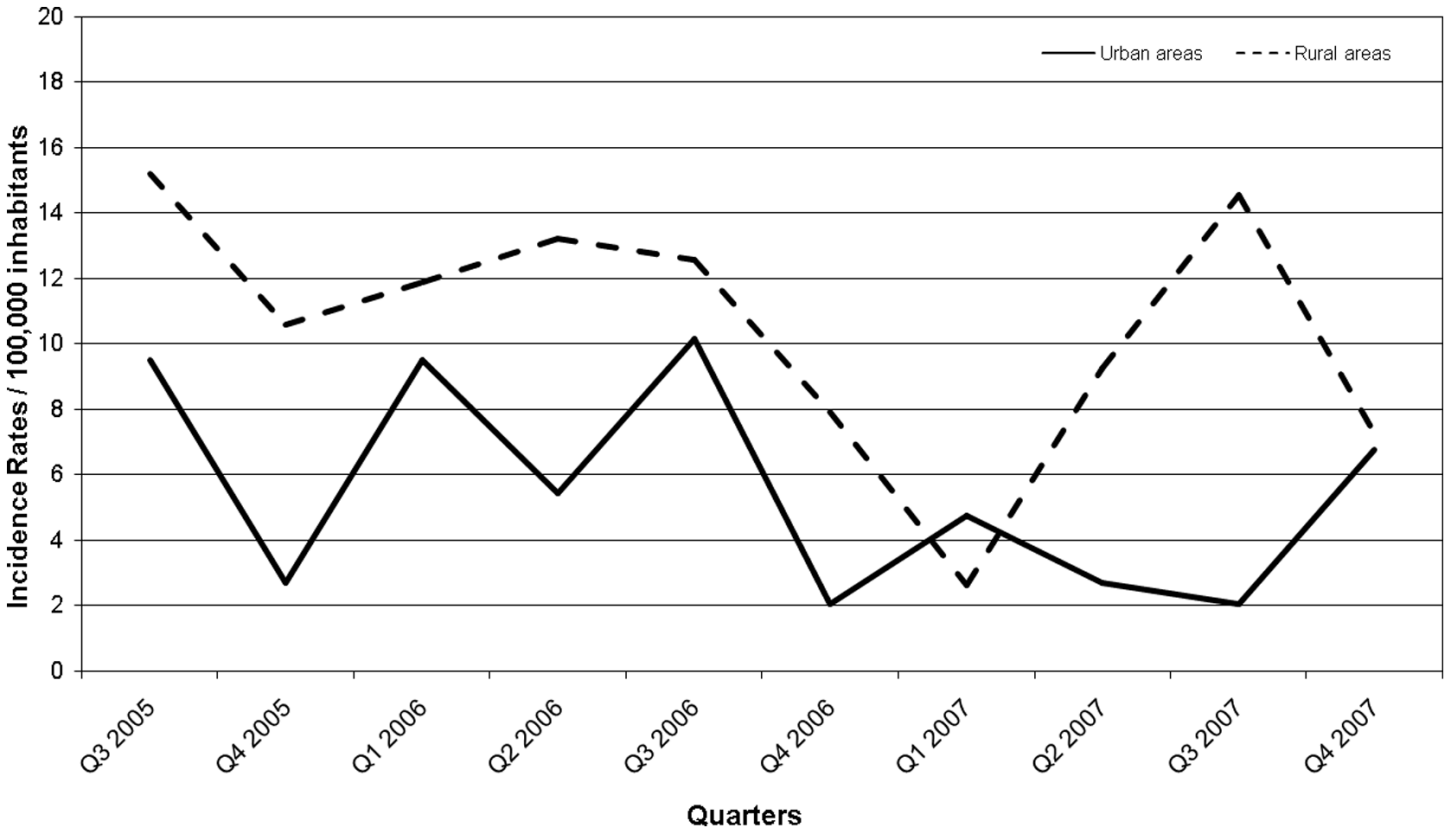
distribution of incidence rates of *Campylobacter* infections from July 2005 to December 2007 for urban and rural areas.

During the study period, the cumulative crude incidence rate of campylobacteriosis was 117.2/100,000 inhabitants in the Eastern Townships, compared to 80.6/100,000 inhabitants in the remainder of Quebec province (*p* = 0.01). The annual incidence rates decreased during the study period from 39.2/100,000 inhabitants over the first 12 months to 26.1/100,000 inhabitants over the following 12 months (*p = *0.0052) (Table S1). Seasonal variation in incidence rates, with a peak during the third calendar quarter (i.e., July, August, and September) was more consistent among rural cases (Figure 1). The overall rate of *Campylobacter* infections was significantly higher in the first quarter (i.e., January to March) of 2006 compared to the same period in 2007 (*p* = 0.0004).

Among the 89 municipalities, 72 were categorized as rural areas, one as a small metropolitan area (hereinafter called urban area), one as a small non-metropolitan city area, and 15 as small town areas; the latter two categories were combined and defined as semi-rural areas. The cumulative incidence rates varied from 55.6/100,000 inhabitants in the urban area, to 110.7/100,000 in the semi-rural area and 96.6/100,000 in the rural area; this difference was observed for both years of the study (data not shown). The risk of campylobacteriosis in rural and semi-rural areas was 1.89-fold greater than in the urban area (*p*<0.0001). Since the incidence rates (both global and by age group) were similar in the rural and semi-rural areas, these two categories were combined and hereafter are referred to as rural areas. The population of the pooled rural areas (n = 151,255) was very similar to that of the urban area (n = 147,430).

**Figure 2:**
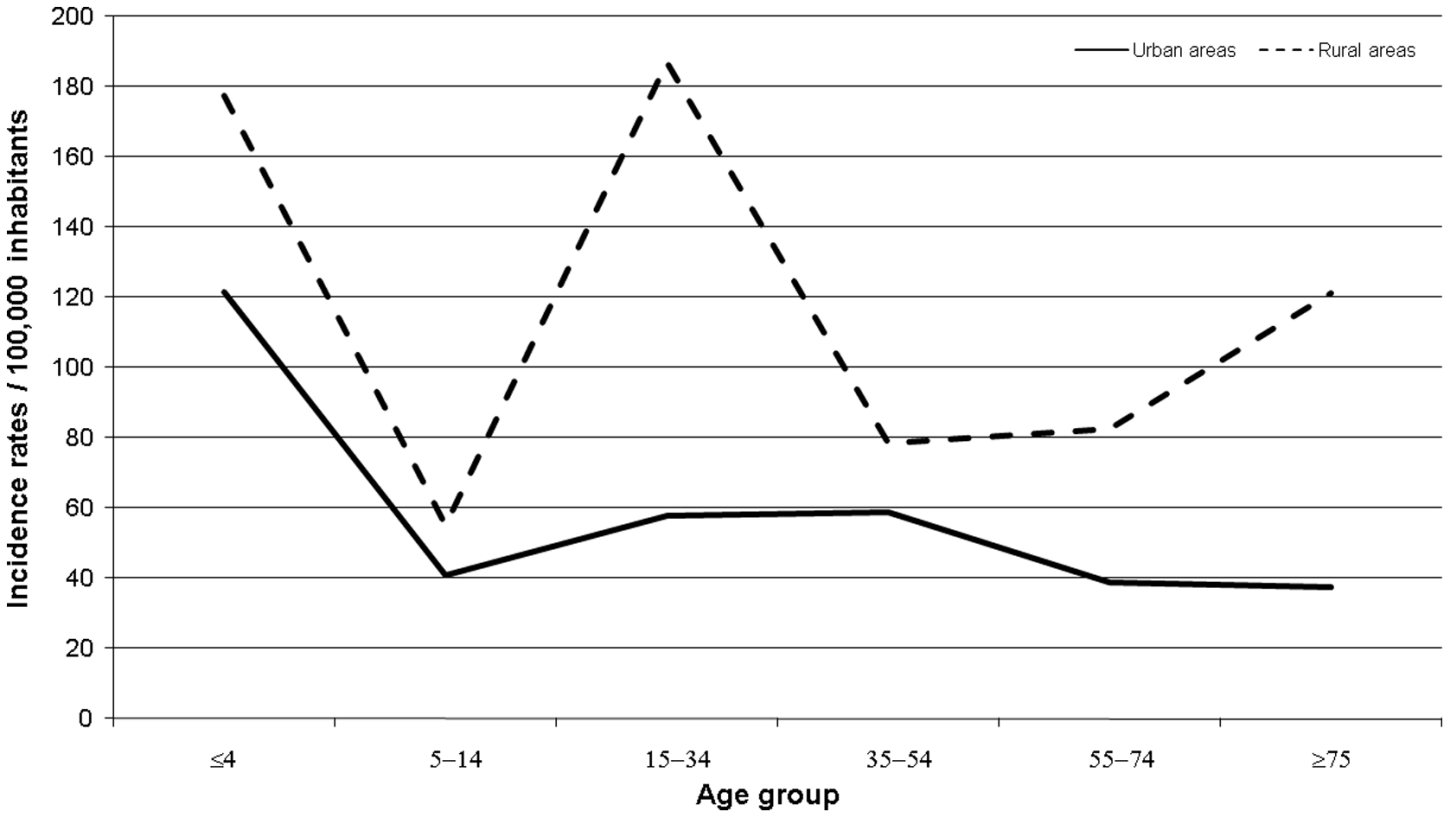
Distribution of incidence rates of *Campylobacter* infections by age group for urban and rural areas.

**Figure 3:**
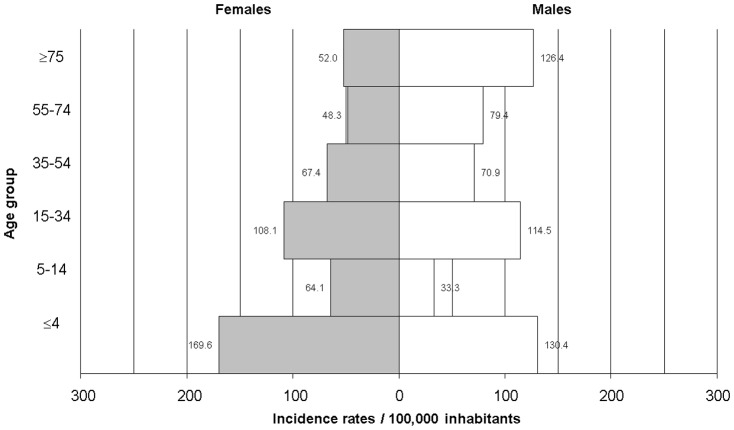
Incidence rates of *Campylobacter* infections in the Eastern Townships by age group and sex.

The median age of the cases was 34 years (range: 9 months to 85 years) and 116 (48.1%) cases were female. The incidence rates of campylobacteriosis varied considerably by age (Figure 2) and by sex (Figure 3 and Figure S1). Overall, the incidence rate among 0–4 year-old children was 149.2/100,000, which was significantly higher than that of the general population as a whole; this was true for both urban and rural areas. In the urban area, incidence rates for all other age groups were similar, ranging from ∼40 to 60/100,000. In contrast, in rural areas, incidence rates were significantly higher among people aged 15–34 years (186.8/100,000) (compared to urban area, IRR = 2.8; 95% CI: 1.6–4.9; *p* = 0.0001), and among those aged ≥75 years (120/100,000) (compared to urban area, IRR = 3.2; 95% CI: 1.0–13.56; *p* = 0.0305).

#### Case-case comparison

Of the 45 exposure factors tested univariately by logistic regression analysis (adjusted for age and sex), seven were significantly more frequent (*p*<0.05) among the cases that occurred in rural areas compared to those that occurred in urban areas (Table 2). Only one exposure (drinking bulk water) was significantly less frequent among cases that occurred in rural areas (OR = 0.5, *p* = 0.04). There were no significant differences in activities related to consuming or handling poultry between urban and rural cases. Of note, 81% of rural cases who reported drinking filtered water (either directly at the faucet or in a pitcher) received their household water from a private well. Univariate analyses restricted to the 15–34 year-old age group showed that factors such as consumption of household water from a private well (OR = 30.2, 95% CI: 5.1–177.0, *p*<0.0002), and living close to a farm (OR = 9.2, 95% CI: 1.8–46.6, *p* = 0.007), were significantly more frequent among the cases occurring in rural areas compared to those occurring in urban areas. However, in the same age group, consuming undercooked chicken (OR = 0.2, 95% CI: 0.3–0.7, *p* = 0.018) was significantly less frequent among the cases acquired in rural areas compared to those acquired in urban areas.

**Table 2:** Comparison of exposure factors for human campylobacteriosis in rural vs urban areas.

					Univariate analysis: rural versus urban area		
Risk factors	Rural area		Urban area		Adjusted for age and sex		
	Cases	Rates (%)	Cases	Rates (%)	OR	95% CI^a^	p value
Living on a farm	39/155	25.2	2/78	2.6	13.3	3.1–56.8	0.0005
Working in a petshop, farm, zoo or veterinary clinic	17/135	12.6	1/69	1.5	11.4	1.5–88.9	0.0201
Household water from a private well	92/151	60.9	10/75	13.3	10.0	4.8 – 21.1	<0.0001
Living close to a farm	57/157	36.3	3/79	6.2	9.2	3.5–24.2	<0.0001
Not having disinfected the private well in the last 6 months	77/88	87.5	6/10	60.0	5.0	1.2 – 21.1	0.0298
Consuming filtered water	32/157	20.4	6/81	7.4	3.2	1.3–8.1	0.0123
Consuming raw milk	13/48	21.3	6/82	7.3	2.9	1.1–7.5	0.0288
Consuming bulk water	17/151	11.3	18/82	22.0	0.5	0.2 – 0.9	0.0414

Risk factors	Multivariate analysis adjusted for age and sex: rural versus urban area		
	OR	95% CI	p value
Working in a petshop, farm, zoo or veterinary clinic	10.6	1.2 – 91.0	0.0320
Household water from a private well	8.3	3.4–20.4	<0.0001

a 95% CI: 95% confidence interval for the odds ratio (OR).

Multivariate logistic regression analysis identified two independent risk factors associated with human *Campylobacter* infection in rural area (Table 2): professional exposure to animals (pet shop, farm, zoo, or veterinary clinic) (OR = 10.6, 95% CI: 1.2–91.0, *p* = 0.032) and consumption of household water from a private well (OR = 8.3, 95% CI: 3.4–20.4, *p*<0.0001).

### Animal and environmental isolates

**Figure 4:**
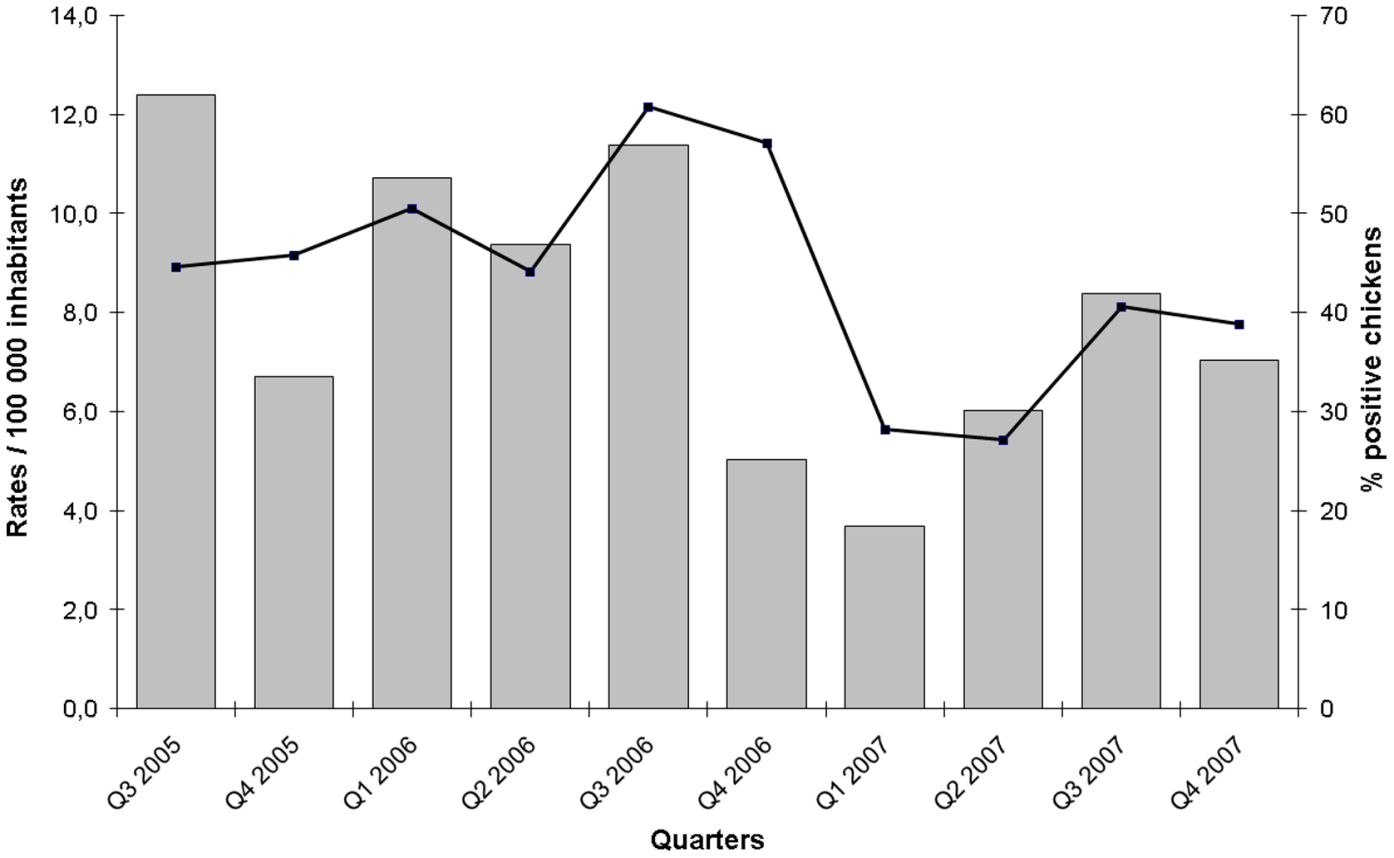
Quarterly distribution of the incidence rates of *Campylobacter* infections in humans from July, 2005 to December, 2007 (columns) and of the prevalence of *Campylobacter* in whole retail chickens from July, 2005 to October 2007 (line graph).

A total of 879 chickens from 59 different food stores were examined for *Campylobacter* presence through culture, and 371 (42%) of them yielded *Campylobacter*. The culture rates were not significantly different for chickens purchased in grocery stores from urban and rural areas (*p* = 0.7). The distribution of *Campylobacter* species among the isolates obtained from chicken was similar to those obtained from human campylobacteriosis cases: 89% of isolates were *C. jejuni* and *C. coli* isolates were the next most common species among isolates. There was no clear seasonal variation for the frequency of culture-positive chickens (*p* = 0.246). The positivity rates of *Campylobacter* in chickens appeared to decrease over the course of the study (Figure 4). These positivity rates correlated modestly with the campylobacteriosis incidence rates in humans (Spearman's ρ coefficient = 0.27; *p* = 0.008) (Figure 4).

Of the 485 bovine fecal samples, 174 (35.9%) were positive for *Campylobacter*, with isolates recovered significantly more frequently from dairy cattle (39.2%) than from beef cattle (20.8%) (p = 0.0001). The distribution of *Campylobacter* species in bovine isolates was somewhat different from that observed in human or chicken isolates (Table 1). Of note, *C. jejuni* was significantly less frequent in beef cattle (48.3%) compared to dairy cattle (77.2%, *p* = 0.001), while the opposite was true for *C. fetus* (41.4% in beef cattle and 15.9% in dairy cattle, *p* = 0.002).

Of the 639 wild bird feces sampled, 108 (16.9%) were positive for *Campylobacter*. Snow geese had the highest positivity rate with 13 (46.4%) isolates, followed by gulls with 78 (33.2%) isolates, Canada geese with 6 (25%) isolates and ducks with 11 (3.1%) isolates. Table 1 shows the overall distribution of *Campylobacter* species in wild birds. *C. jejuni* was the most prevalent species in each of the bird species surveyed and the only one present in ducks. *C. lari*, *C. fetus* and *C. upsaliensis* were isolated only from gulls.

### Multi-locus sequence typing

**Figure 5:**
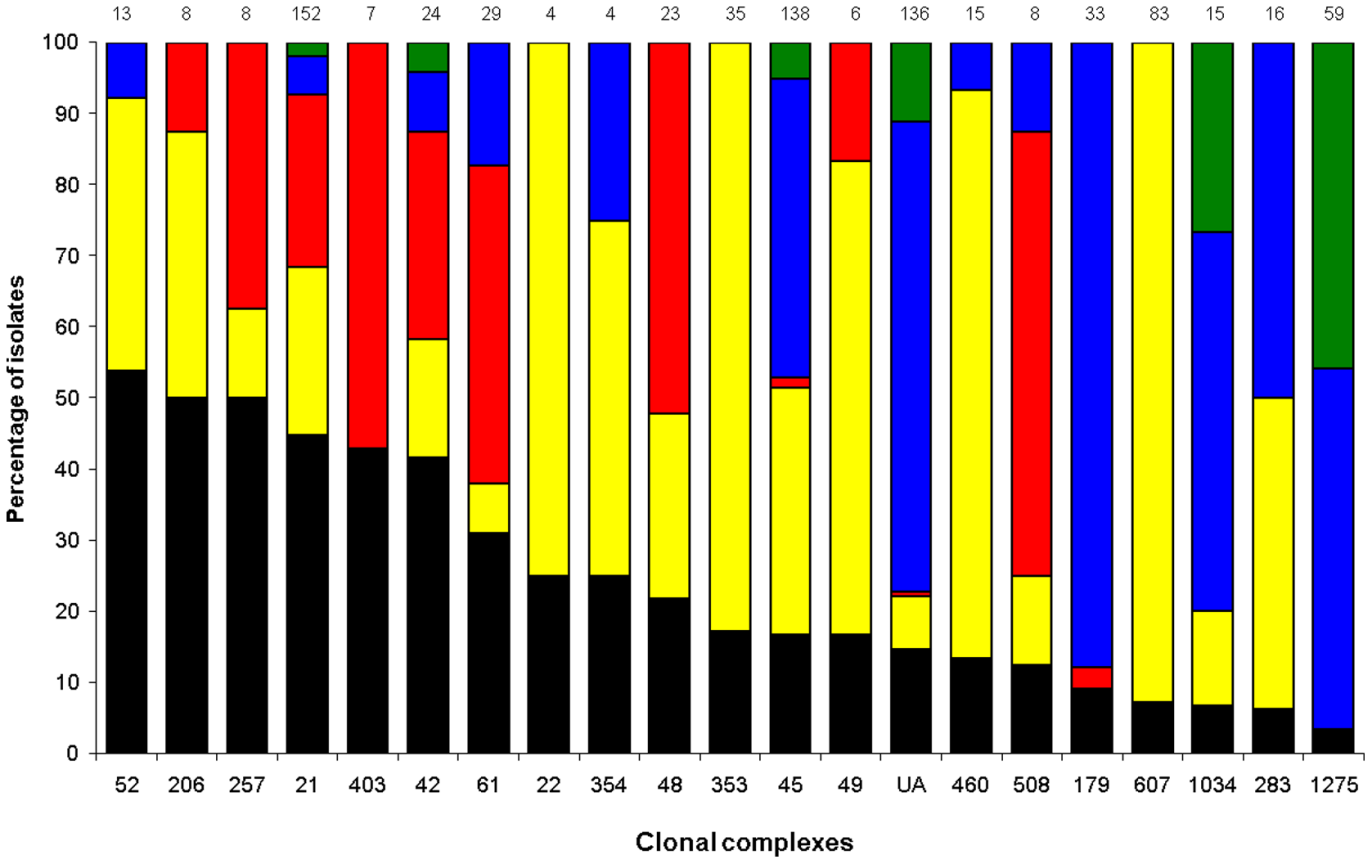
Distribution of attributable sources of human *C. jejuni* isolates by clonal complexes. Only clonal complexes found in human isolates are represented. Human isolates are in black, chicken isolates are in yellow, bovine isolates are in red, water isolates are in blue and wild bird isolates are in green. The remaining 108 isolates were distributed among 74 STs which could not be assigned to any of the known lineages (unassigned STs; UA). Numbers at the top of the columns indicate the total number of isolates in each clonal complex.

A total of 851 *C. jejuni* isolates (178 human, 257 chicken, 87 bovine, 266 water and 63 wild bird isolates) were typed by MLST. Of the 262 STs identified (Table S2), 188 STs, accounting for 743 (87%) isolates, were assigned to 31 previously described CCs. The remaining 108 isolates were distributed among 74 STs which could not be assigned to known lineages. The most frequent STs identified were ST-45 (96 isolates; 11.3%), ST-1212 (74 isolates; 8.7%) and ST-21 (66 isolates; 7.8%). Overall, 204 STs (77.9% of the STs) were represented by only one or two isolates in the collection. The most prevalent CCs identified were CC-21 (152 isolates), CC-45 (138 isolates) and CC-607 (83 isolates); together these represented 43.8% of typed isolates. Only three CCs (CC-21, CC-45, and CC-42) were found in humans and in all four sources (Figure 5); however, 20 (65%) of the 31 CCs observed in this study included isolates from humans and at least one of the sources.

Among the 262 STs identified, 160 (61.1%) were previously unreported in the international database, although 108 (67.5%) of them could be assigned to known CCs (Table S2). Among the new STs, 43 (26.9%) resulted from new allele sequences, and the remainder from new combinations of previously described alleles (data not shown). Of note, three water isolates had new allele sequences detected in all seven genes; one isolate had a deletion at position 461 of the *aspA* allele and was not submitted to the international database [24]. Most (87.5%) of the new STs were represented by a single isolate. New STs were most common among water isolates (93/266 isolates; 34.9%), followed by isolates from wild bird (18/63 isolates; 28.6%), human cases (19/178 isolates; 10.7%), chicken (27/257 isolates; 10.5%), and cattle (3/87 isolates; 3.5%). Genetic diversity was measured at the ST level among sources using the Simpson's index of diversity (D). Isolates from each sources were characterized by highly diverse genetic lineages (chicken isolates; D = 0.89, bovine isolates; D = 0.91, wild bird and human isolates; D = 0.95, and water isolates; D = 0.97).

Among the human isolates, 113 rural and 65 urban isolates were typed by MLST. The four most frequent CCs identified among these human isolates were CC-21 (38.2% of the typed human isolates), CC-45 (12.9%), CC-42 (5.6%) and CC-61 (5.1%). The incidence rates of these CCs were analyzed by area type and age (Table 3). The rate of CC-61 was significantly higher in rural than urban areas (ratio 7.8; 95% CI: 1.1–346.0). While the overall rate of CC-21 was higher in rural areas (ratio 1.6; 95% CI: 0.9-2.7), this difference was statistically significant in those aged 15–34 years (ratio 2.6; 95% CI: 1.3–5.7). When each of the exposures was analyzed by CC, significant associations were found only for CC-61, which was associated with occupational exposure to animals (*p* = 0.03) and with consuming raw milk (*p* = 0.003). Among patients with occupational exposure to animals, 16 worked on bovine farms, one in a poultry farm and one in a veterinary clinic; 13 belonged to the 15–34 year-old age group.

**Table 3:** Proportion of CCs from human isolates among age groups and areas.

Clonal complex	Age group	Isolates		Incidence rate per 100,000		Incidence rate ratio	95% incidence intervals	p value
		Rural area	Urban Area	Rural area	Urban area			
ST-21	0–14	8	6	31.4	24.5	1.3	0.4 – 4.5	-
	15–34	25	12	75.8	28.9	2.6	1.3 – 5.7	0.004
	35–54	7	5	15.3	11.8	1.3	0.4 – 5.2	-
	≥55	2	3	4.3	7.7	0.6	0.1 – 4.8	-
	Total	42	26	27.8	17.6	1.6	0.9–2.7	-
ST-45	0–14	0	3	0.0	12.3	-	-	-
	15–34	2	0	6.1	0.0	-	-	-
	35–54	5	3	10.9	7.1	1.6	0.3–10.0	-
	≥55	8	2	17.0	5.1	3.3	0.7–32.0	-
	Total	15	8	9.9	5.4	1.8	0.7–5.0	-
ST-42	0–14	0	0	0.0	0.0	-	-	-
	15–34	2	1	6.1	2.4	2.5	0.1–148.5	-
	35–54	2	0	4.4	0.0	-	-	-
	≥55	2	3	4.3	7.7	0.6	0.1–4.8	-
	Total	6	4	4.0	2.7	1.5	0.4–7.0	-
ST-61	0–14	3	0	11.8	0.0	-	-	-
	15–34	4	0	12.1	0.0	-	-	-
	35–54	0	1	0.0	2.4	-	-	-
	≥55	1	0	2.1	0.0	-	-	-
	Total	8	1	5.3	0.7	7.8	1.1–346.0	0.02
UA	0–14	3	2	11.8	8.2	1.4	0.2–17.3	-
	15–34	4	2	12.1	4.8	2.5	0.4–27.8	-
	35–54	2	3	4.4	7.1	0.6	0.1–5.4	-
	≥55	4	0	8.5	0.0	-	-	-
	Total	13	7	8.6	4.7	1.8	0.7–5.4	-

**Figure 6:**
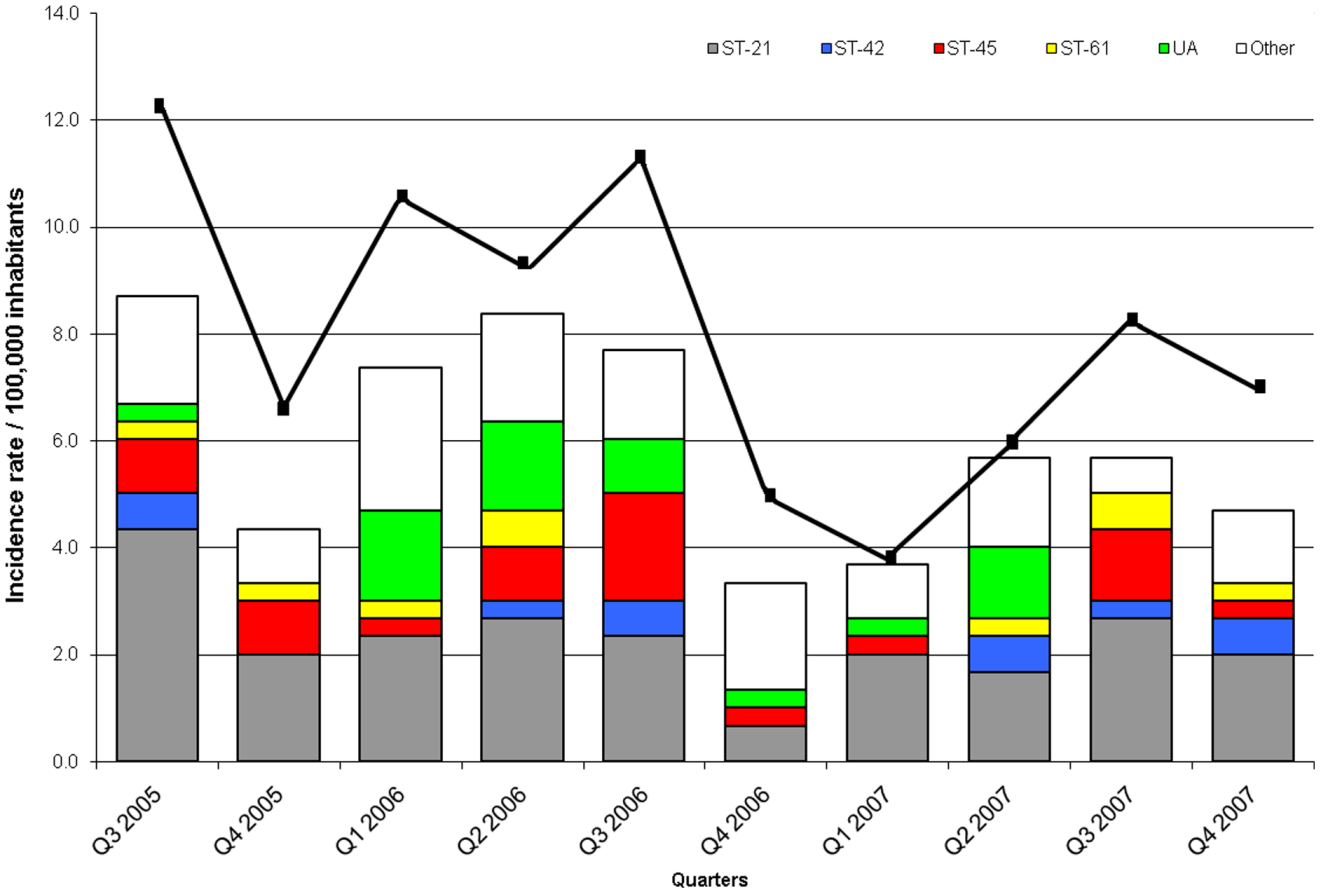
Quarterly distribution of clonal complexes (in colors) among human isolates typed by MLST. Incidence rates of *Campylobacter* infections in the Eastern Townships is indicated by the black line. UA: unassigned STs to a clonal complex.

Analysis of the distribution of the CCs in human isolates throughout the study period indicated that CC-21 was present in the same proportion all over the period, while CC-45 and CC-42 contributed strongly to the third quarter seasonal peak (Figure 6). There appeared to be a trend for unassigned ST isolates to be prominent in the second quarter of the year.

### Source attribution of human campylobacteriosis

All CCs found in human isolates were also found in at least one of the sources (Figure 5). Of interest, for some CC a single source was dominant or even exclusive. For example, CC-607 and CC-353 were exclusively associated with chicken, CC-179 predominantly with water and CC-61 predominantly with bovine.

**Figure 7:**
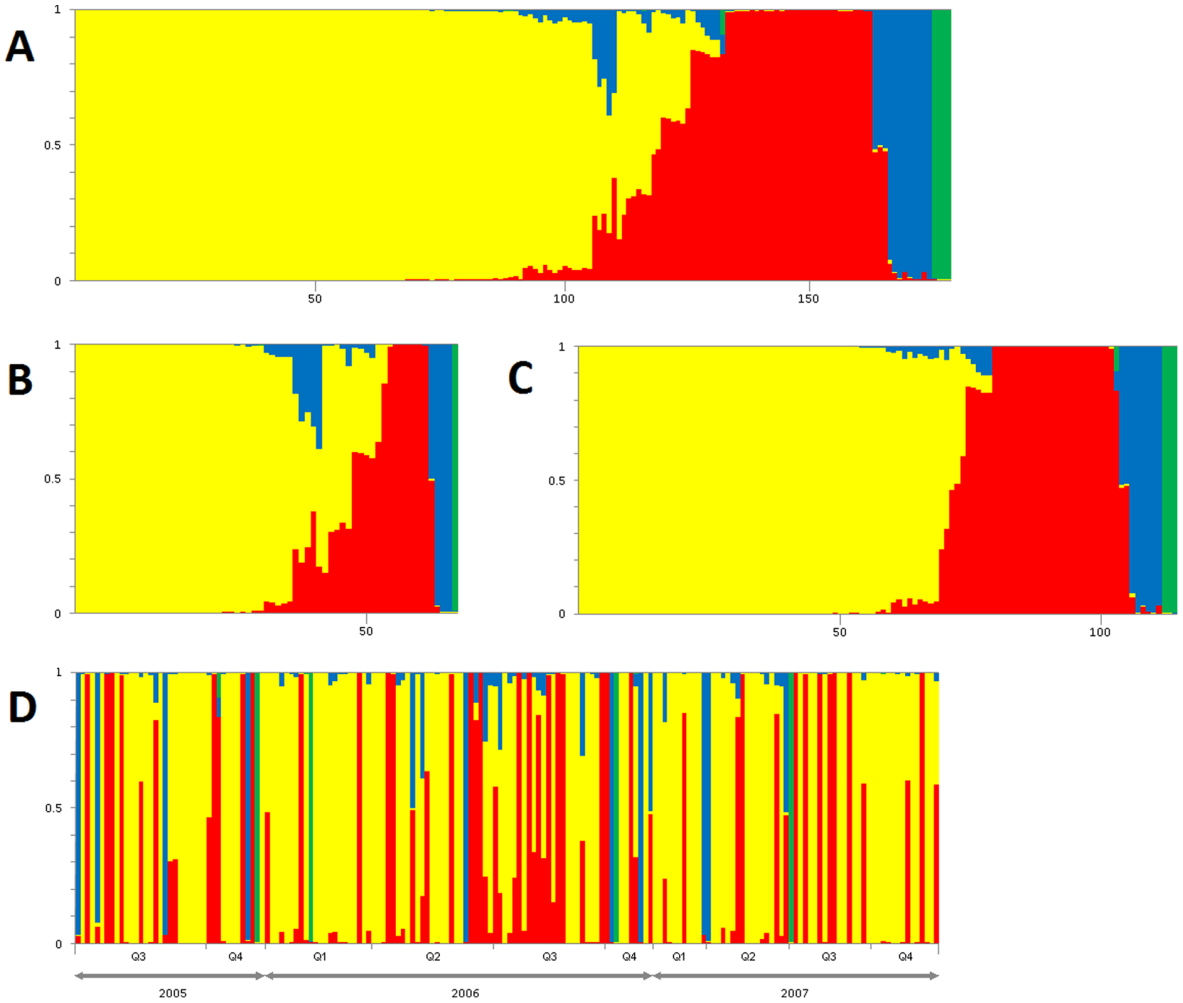
The source probability assignment (y axis) of human campylobacteriosis cases (x axis) using the no-admixture model of STRUCTURE. Each isolate is represented by a vertical bar, showing the estimated probability that it comes from each of the putative sources. Sources for *Campylobacter jejuni* were chickens (yellow), cattle (red), water (blue) and wild birds (green). Panel A showed all the 178 human cases typed, panel B cases from urban area and panel C from rural area. Isolates in panel A, B and C are ordered based on probability of source using the hierarchy chicken, bovine, water and wild bird. Panel D showed all the human cases sorted in the time by quarter of positive sampling.

The assignment probability for each putative source was calculated for each human isolate individually (Figure 7) and the percentage of all human isolates attributed to each source was calculated as the average of these probabilities. Among the human *C. jejuni* isolates analyzed, 64.5% (95% CI: 58.0–71.0%) were attributed to chicken, 25.8% (95% CI: 20.0–31.6%) to bovine, 7.4% (95% CI: 4.1–10.7%) to water and 2.3% (95% CI: 0.1–4.5%) to wild birds. Attribution estimates for the four putative sources were similar in both rural and urban areas. Only cases aged 15–34 years were significantly more frequently associated with cattle in rural than urban areas (19.5% vs 8.2%, *p = *0.02). Among the sources, only cattle showed a clear seasonal variation, since the majority of the cases were found during summer and fall, particularly in 2006 (Figure 7). Human cases associated with chicken were equally distributed all over the year.

## Discussion

We investigated the sources of human campylobacteriosis in the Eastern Townships of Quebec during a 30-month period and used MLST to characterize the *Campylobacter* isolates from human cases and from the contemporaneous samples of retail fresh whole chickens, bovine and wild bird feces, and environmental water. We identified rurality of residence location, age, season, and exposures such as occupation with animals and household water coming from a private well as important risk factors for human campylobacteriosis. The combination of clinical and molecular analyses yielded several novel insights, provided more precise estimates of the particular sources of sporadic human campylobacteriosis, and identified specific CCs represented among the human isolates that were associated exclusively or predominantly with particular sources.

### Chicken as a source of human campylobacteriosis

Our study confirms that chicken is the most important source of human campylobacteriosis. *Campylobacter* was isolated from over 40% of the retail chickens examined and the species distribution of chicken isolates was similar to that observed among human isolates. MLST identified seven CCs represented among the human isolates for which chicken was the exclusive or predominant putative source. The two most frequent CCs for human cases (CC-21 and CC-45) were also frequently found among chicken isolates. Source attribution analysis indicated that, overall, chicken accounted for 65% of human cases. This is consistent with most of the recent case-control studies of sporadic campylobacterosis [25]–[29] and with source attribution studies conducted in United Kingdom [4], [15], the Netherlands [16] and New Zealand [30]. No significant differences between urban and rural areas, nor among age groups, were found in chicken attributions. This is in contradiction with previous studies in other parts of the world where chicken is a more important source of infection in young urban children than in their rural counterparts, for which ruminant and other avian sources are more important [16], [22], [23]. Besides variations in local epidemiology, such divergences may be due to the limited number of cases in this age group in our study.

### Cattle as a source of human campylobacteriosis

We identified cattle as the second most frequent putative source for human campylobacteriosis, accounting for 26% of overall cases. Among isolates from the four putative sources, bovine isolates represented 65% of the CC-61 isolates. CC-61 ranked fourth in the number of human isolates and was significantly associated with professional exposure to animals and raw milk consumption. In the Eastern Townships, 80% of animal husbandry consist of cattle and only 0.8% are chicken [31]. Farmers, their families and neighbors often consume raw milk. Consumption of raw milk has been previously associated with campylobacteriosis [6], [32], [33]. Highest incidence in the 0–4 age group in agricultural settings has also been reported [7], [21], [34], and has recently been linked to contact with farm animals [26]. Our molecular typing results directly support these connections and confirm that cattle are an important, and often underestimated, reservoir for human Campylobacter infection and that bovine exposure contributes significantly to the increased risk in rural compared to urban areas, particularly in the 15–34 year age group. The fact that dairy cattle had a significantly higher *Campylobacter* prevalence has previously been observed also in a Spanish study in which it was suggested that the use of different husbandry systems might account for these differences [35].

### Water as a source of human campylobacteriosis

Consumption of private well water was the other risk factor associated with residence in rural areas compared to urban areas in the multivariate analysis. Not having disinfected the private well in the past 6 months was a risk factor in the univariate analyses. Especially in rural areas, fecal contamination of wells with pathogenic bacteria may occur by several different mechanisms, including rain runoff of surface water, particularly after flood conditions and/or the distribution of manure sludge to farmland [36]–[41]. Although surface water is more prone to contamination than ground water, ground water is often consumed without monitoring or treatment; consequently, even low levels of contamination can result in appreciably increased risk of infection [42].

Water isolates were represented in 60% of the CCs that included human isolates. In the source attribution analysis, only 7.4% of infections were assigned to water, but this percentage would not include infections already assigned to chicken or cattle. Indeed, cattle-specific *C. jejuni* infections have been associated with waterborne outbreaks [36]. CC-179 was the only CC for which water was the almost exclusive non-human source. In CC-45, the non-human isolates were isolated essentially equally from water and chicken, and in CC-1275, from water and wild birds. Studies of water isolates in northwest England [43] and New Zealand [44] have also found a predominance of CC-45. Surprisingly, of the CCs predominantly associated with cattle, only CC-61 was also found (>15%) in water isolates. The hypothesis that ST-45 is an environmentally well-adapted type which can survive under stress better than other STs [43] is supported by our findings. A recent study also found that isolates from CC-21 and CC-45 had different survival patterns after being submitted to various stresses [45]. However, the low frequency of water isolates among CC-21, the most prevalent human genotype, argues against the hypothesis that human sewage is an important source of *Campylobacter* in river water [44].

### Occupational exposure

Previous studies have identified an increased risk of campylobacteriosis among dairy farm and poultry abattoir workers [46], [47]. In our study, occupational exposure was reported for 18 cases (of which 17 were rural inhabitants, with isolates significantly associated with bovine MLST types) and was one of the only two risk factors that remained significant by multivariate analysis. These findings support the hypothesis of Mullner et al. that bovine-derived cases are typically the result of environmental and occupational, rather than food-borne, exposures, in contrast to poultry-associated cases, where the handling and consumption of undercooked chicken is the dominant transmission pathway [30].

### Seasonal variation

The seasonal variation observed over the 30 months of this study, with a sharp increase from May until October, is similar to that previously described [6], [9], [48], [49]. Three CCs (CC-61, CC-45, and CC-42) showed strong seasonal variation. Among these, CC-45 was well represented among water isolates and the other two among water and bovine isolates. Late spring through early fall coincide with periods of manure spread [50] and the highest water contamination in the Eastern Townships. In other reports, only CC-45 had been associated with increased rural incidence rates [9]. Seasonal variation was more consistent among cases occurring in rural area and MLST results suggested that bovine and water sources contributed to this pattern.

### MLST genotype diversity

Overall the genotypes observed in this study are consistent with prior analyses. The most frequent CCs and STs were similar to those identified in other reports [8]–[10], [51]–[55]. The majority of STs not previously reported represented water isolates and these STs often could not be assigned to a known CC, as well as individual alleles and allele combinations not previously reported. This phenomenon was noted in our earlier study [51] and also by others [43], [44]. CC-45, the second most common CC in feces of wild birds, was the predominant genotype in a study of similar samples [56]. Isolates in our study were characterized by highly diverse genetic lineages (genotypes). We also observed that genotypes from different sources were not randomly distributed, except for CC-21, which was unique in having a balanced representation from both chicken and bovine sources. More interestingly, some non human isolates were never found among human clonal complexes. These observations support the hypothesis that genomic diversity is related to niche adaptation and, consequently, some genotypes may be restricted to specific ecologic niches or even a single host species [57], and some genotypes may not have the capacity to cause symptomatic infection in humans [58]. Empirical studies indicate that some *Campylobacter* isolates are unable to colonize chickens [13], [14], [57] and recent genomic analyses indicate that some strains lack regions required for clinical infection [13].

### Limitations

This study has some limitations. While we sampled four diverse potential sources, we could not assess the contribution of other common animal reservoirs, such as sheep, pigs or pets [4], [15], [16], [59]. Sheep farming is infrequent in the Eastern Townships (6.5% of animal husbandry). Furthermore, as we focussed our study on *C. jejuni* isolates, pigs are not known as the primary reservoir for this species, as pigs are mainly contaminated by *C. coli* [60]. According to case-control studies, contribution of pet ownership to human infection appears not to exceed 10% [26]. Moreover, common sources of infection for pets and humans, and directionality of transmission between pets and humans are to a large extent unknown [61]. However, the contribution of different sources may be influenced by geographic area and could be different in another environmental context.

The comparison of groups of cases with the same disease has the advantages of removing any potential for bias between the compared groups being caused by the selection process of the surveillance system, in addition to the differential recall bias occurring when cases are compared to healthy controls. These comparisons allow for a more restricted but more refined analysis of the association of some exposures with infection. Determination of how exposure to the infectious agent occurred is more efficient and unbiased than in standard case-control studies, but general factors determining whether disease occurs after an infectious exposure cannot be studied [62]. In our study, the combination of the case-case comparison approach with both MLST typing of human, animal and environmental isolates and the STRUCTURE source attribution estimates compensated this limitation.

## Conclusions

In summary, this study examined *Campylobacter* infections using a combination of case-case analysis and molecular strain typing of human, animal and environmental isolates. We validated the findings from these two independent methodologies, to decipher overall source attribution as well as differences between risk factors in urban and rural areas. In our model, chickens were the attributable source for 65% of human campylobacteriosis infections, independently of residential zone, sex and age. The increased incidence in rural compared to urban areas was associated with exposure to cattle, particularly among people aged of 15–34 years, and was significantly related to occupation and water consumption from a private well. Both bovine and water exposure probably contribute to human campylobacteriosis seasonality. These results could be used to develop public education and other preventive programs that target the main risk factors.

## Materials and Methods

### Clinical and epidemiological data

The Eastern Townships comprise seven counties with 89 municipalities totalling 298,685 inhabitants. Each municipality was categorized as a small metropolitan area (50,000–249,999 inhabitants), a small non-metropolitan city area (20,000–49 999 inhabitants), a small town area (2,500–19,999 inhabitants) or a predominantly rural area (<2,500 inhabitants), as defined by Statistics Canada (www.statcan.gc.ca), using population data from the 2006 Canadian Census.

Microbiology laboratories in the Eastern Townships are obligated to report all diagnosed *Campylobacter* infections to the regional Public Health Department as campylobacteriosis is a notifiable disease. Public health nurses interviewed cases by telephone within 2 weeks of reporting, using a structured questionnaire to collect demographic and clinical data, travel history, food history, water consumption, recreational water activity, and animal exposure during the 10 days before the onset of symptoms. All cases reported between July 1, 2005 and December 31, 2007 were eligible for the study. Since interviews were made by phone, oral consent, rather than written consent, was obtained to use the anonymized data from the questionnaire. Public health nurses did not compile the data if oral consent was not obtained and the oral consent was indicated on each questionnaires. Cases were excluded if the infection was acquired outside Quebec (i.e., travel outside the province during the entire 10-day period before the onset of symptoms), if the home address was outside the Eastern Townships, or if they declined to participate. Only the first episode of infection was considered for subjects reported on multiple occasions during the study period. Campylobacteriosis data for the whole Quebec province used to calculate the provincial incidence rate (other than the study area) were provided by the Ministère de la Santé et des Services Sociaux du Québec. This study, including the oral consent procedure, was approved by the ethics committee of the centre de recherche clinique Étienne-Le Bel du centre hospitalier universitaire de Sherbrooke.

### Isolation and identification of *Campylobacter* isolates

In the Eastern Townships, all *Campylobacter* cultures for human stools are done in hospital laboratories at no charge to the patient or prescribing physician. Hospital microbiology laboratories were asked to provide all *Campylobacter* isolates cultured during the study period. The laboratories routinely search for *Campylobacter* in human stool specimens using similar isolation techniques (Karmali or Skirrow media incubated for 72 h at 42°C in a microaerobic atmosphere) and identify *Campylobacter* isolates to the species level by conventional phenotypic methods [63]. Species identification was taken as reported from the laboratories; for isolates reported only as *Campylobacter* spp., species identification was performed by CPN60 gene analysis [24].

In parallel, a prevalence study of environmental (surface) water (collected from 13 rivers and 12 streams in the seven counties of the Eastern Townships), retail fresh whole chicken and two animal sources (bovine and wild bird feces) was performed in the Eastern Townships. River water samples were collected from July, 2005 to October, 2007 [50] and processed as previously described [64]. From May 22, 2005 to October 16, 2007, 8 fresh, eviscerated whole chickens were purchased weekly in the different counties (one chicken per store). A total of 879 chickens were examined for the presence of *Campylobacter*. Of note, retail chickens sold in the Eastern Townships are produced by multiple companies based elsewhere in Quebec Province. From November 11, 2005 to December 11, 2007, 495 samples of fresh bovine feces (365 dairy cattle and 130 beef cattle) were collected from 99 farms distributed among the seven counties of the Eastern Townships. Each farm owner gave permission to conduct the study on their farm. From May 10, 2005 to November 14, 2007, 235 samples of fresh gull feces (round-billed gull [*Larus delawarensis*] and great black-backed gull [*Larus marinus*]) were collected at two waste management sites located in the Eastern Townships. In addition, from May 10, 2005 to November 14, 2007, samples of fresh bird feces were collected from 352 ducks (mallard [*Anas platyrhynchos*]), 28 snow geese [*Chen caerulescens*], and 24 Canada geese [*Branta canadensis*]). Detailed culture methods are presented in the supplementary material.

*Campylobacter* isolates from water, chicken, bovine, and wild bird samples were identified at the species level by routine phenotypic methods as described previously [50]. Genomic DNA was extracted by transferring a single colony of each isolate into 25 µl of 0.5 N NaOH, incubating for 5 minutes at room temperature, and then adding 25 µl of Tris 1 M pH 8.0 and 450 µl of sterile distilled water. DNA extracts were stored at −20°C. Isolates that had a hippurate-negative phenotype but had a hippurate gene detected by PCR were identified as *C. jejuni* [65]. Species identification of *Campylobacter* other than *C. jejuni* was also confirmed by CPN60 gene analysis [24]. Isolates which died before completing species identification were designated as *Campylobacter* spp.

### Multilocus sequence typing

Among the 219 human *C. jejuni* cases included in the study, 178 (81%) isolates were available for MLST typing. Typed isolates from other sources were evenly distributed among sampling periods and sampling areas. MLST was performed either by the high-resolution melting (HRM) system previously described [66] or by the conventional method as described by Dingle et al. [10] with modified amplification conditions [51]. For some isolates, primers of the extended MLST system [67] or new primers designed in a previous study [24] were used. Sequences were compared and analyzed with BioNumerics program version 5.0. Allele numbers, sequence types (STs) and clonal complexes (CCs) were assigned by submitting DNA sequence to the *C. jejuni* MLST database website (http://pubmlst.org/campylobacter).

### Data analysis

Minimum spanning trees were constructed within BioNumerics, using the allelic data set. New STs not assigned to a CC within the *C. jejuni* MLST database were examined for CCs by use of eBURST3 [68], [69]. As proposed by Dingle et al.[10], a CC was defined as two or more independent isolates with STs that shared identical alleles at four or more loci. CCs were constructed using a maximum neighbor distance of two changes and a minimum size of two STs. Genetic diversity was measured using the Simpon's index of diversity [70].

The probability for each human isolate in the study to be attributable to the putative sources examined was estimated by comparing its MLST profile (based on sequence data) to the reference set comprising the genotypes of the contemporaneous animal and environmental isolates. This analysis was performed using STRUCTURE, a Bayesian clustering method that uses multilocus genotype data to infer population structure and assign individuals to populations [71]. Differences in genotype frequencies among populations in the reference data set allow probabilistic assignment of isolates to a population, even for genotypes shared among the populations. This model has been demonstrated to provide epidemiologically useful estimates of the likely origin of a clinical isolate [71]. Analyses were performed with 10,000 iterations following a 10,000-iterationburn-in using the no-admixture model of STRUCTURE; the human isolates to be assigned were distinguished from the reference data set isolate populations using the “usepopinfo” flag.

### Statistical analysis

Incidence rates of human campylobacteriosis for each area were compared by Chi-square test or Fisher's exact test. For the case-case analysis, we compared the incidence rate ratios (IRR) and the risk factors for campylobacteriosis among patients living in rural vs urban areas, as defined in the Results Section, and 95% confidence interval (CI) for the IRR was calculated. Risk factors for campylobacteriosis were examined using unconditional logistic regression analysis. All regression models included age and sex as covariates. Age was used as a continuous variable. Risk factors with *p*<0.10 at the univariate analysis were included in a multivariate logistic regression model built through stepwise forward selection. Correlations between independent variables were assessed for collinearity in the multivariate model using Spearman's ρ statistic. All these correlations were less than 0.3 and there were no significant association between the independent variables. Association between chicken positivity rates to *Campylobacter* and human campylobacteriosis incidence rates was tested for significance with Spearman's ρ statistic. Seasonal differences in chicken positivity rates to *Campylobacter* were tested for significance with Kruskall-Wallis test for independent samples. Differences between STRUCTURE attribution estimates between sources were tested for significance with the Mann-Whitney U test for independent samples. Significance level for all analysis was *p*<0.05. All statistical analyses were performed using SAS version 18 (SAS, Cary, NC).

## Supporting Information

Figure S1 Incidence rates of *Campylobacter* infections by age group and sex. For urban area (A) and for rural area (B).
(TIF)

Methods S1 (DOC)

Results S1 (DOC)

Table S1 Incidence rates of campylobacteriosis in each county and for each municipality category in the Eastern Townships
(DOC)

Table S2 Distribution of 851 *C. jejuni* isolates among clonal complexes, sequence types (ST) and isolation sources. New STs identified in this study and in our previous study (2) are in boldface and new CCs are italicized.
(DOC)
