# Supplementary figures and images for: Campylobacteriosis in Urban versus Rural Areas: A Case-Case Study Integrated with Molecular Typing to Validate Risk Factors and to Attribute Sources of Infection
Source: PLoS One. 2013 Dec 26;8(12):e83731. doi: 10.1371/journal.pone.0083731 (PMC3873381; doi:10.1371/journal.pone.0083731)

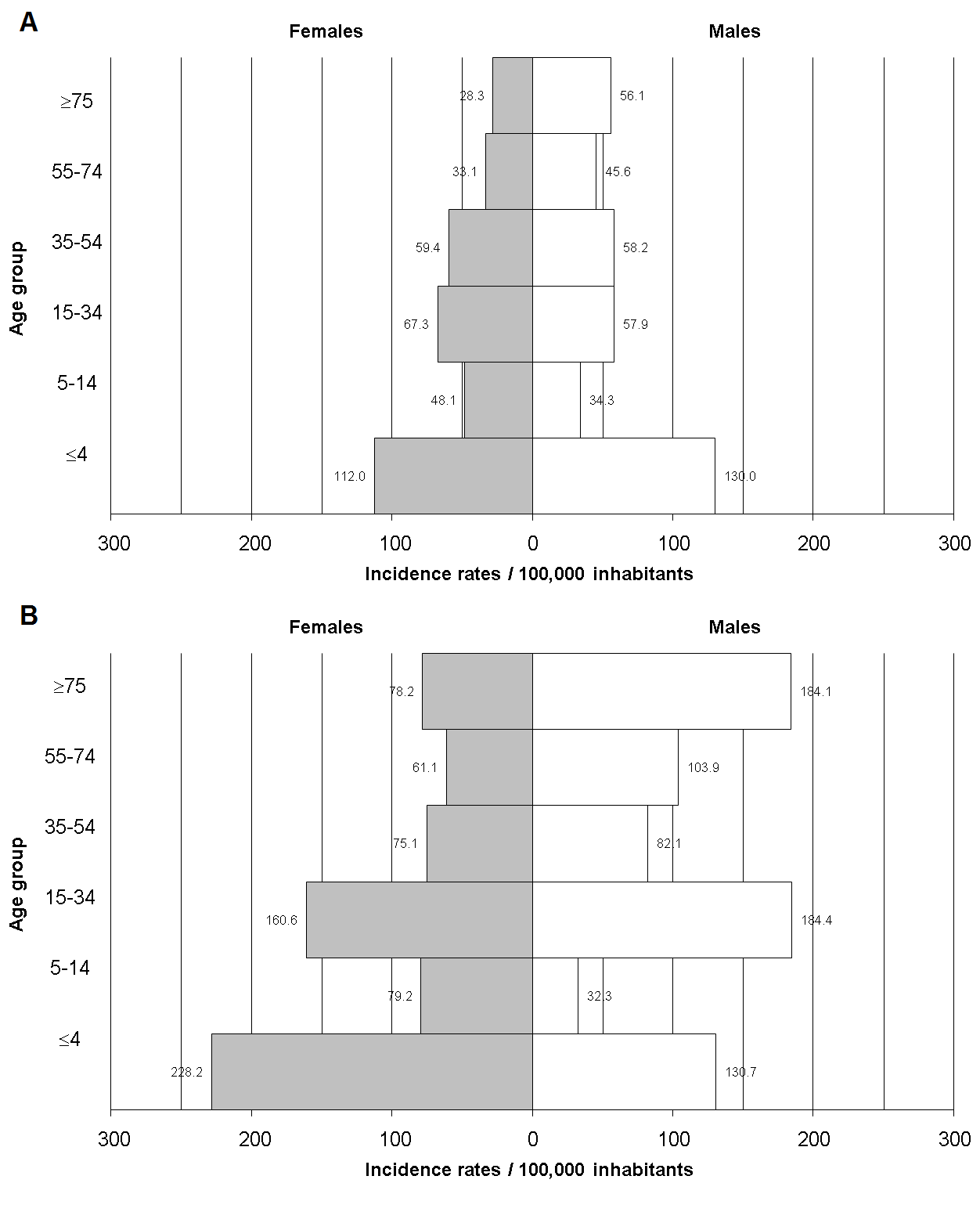

Supplement: Figure S1 — Incidence rates of Campylobacter infections by age group and sex. For urban area (A) and for rural area (B). (TIF) [file pone.0083731.s001.tif]
