# Supplementary material for: Campylobacteriosis in Urban versus Rural Areas: A Case-Case Study Integrated with Molecular Typing to Validate Risk Factors and to Attribute Sources of Infection
Source: PLoS One. 2013 Dec 26;8(12):e83731. doi: 10.1371/journal.pone.0083731 (PMC3873381; doi:10.1371/journal.pone.0083731)
Supplement: Methods S1 — (DOC) [file pone.0083731.s002.doc]

**Supplemental material**

MATERIAL AND METHODS

**River water analysis.** We identified 32 sampling sites on 13 rivers and 12 streams across the 7 counties of the Eastern Townships, Quebec, Canada and sampled weekly from July 17, 2005 to November 25, 2007. For each sampling site, ~ 3000 ml of water were collected weekly from the nearshore areas or from a bridge passing over the site, using a horizontal alpha water sampler (Geneq, Montreal, Canada) at a depth about 15 to 30 cm below the surface. The sample was transported on ice to the laboratory, held at 4°C and tested within 24 h. Water was filtered through a 0.45 µm pore-size membrane filter and Preston selective enrichment broth (Oxoid, Nepean, Ontario, Canada) and Karmali agar (Oxoid) were used to isolate *Campylobacter* .

**Retailed whole fresh chicken analysis.** The chickens were stored at 4°C overnight and washed vigorously with 250 mL of nutrient broth. The broth was filtered through cheesecloth and centrifuged at 16,300 x g for 15 min. The sediment was suspended in 5 mL of Brucella broth (Oxoid); 100 mL of Preston broth were added to the suspension, gently mixed, and incubated under microaerobic atmosphere at 37°C for 4 h, then at 42°C for 48 h. Next, 200 µl of the suspension were plated on Karmali agar and incubated at 42°C for 48 h under microaerobic conditions.

**Bovine feces analysis.** Feces samples were transported in Enteric Plus medium (Meridian Bioscience Inc, Ohio, USA) and processed on the same day. About 1-2 g of each feces sample were transferred to 25 ml of Preston broth and incubated 3-4 h at 37°C and then transferred to 42°C to complete 48 h of incubation. After incubation of this enrichment broth, 200 µl were streaked on a Karmali plate and incubated at 42°C for 48 h.

**Wild bird feces analysis.** Fresh feces samples picked from the soil (gulls, Canada gooses and white gooses) were transported in Enteric Plus medium and processed on the same day. About 1-2 g of each feces sample were transferred to 25 ml of Preston broth and incubated 3-4 h at 37°C and transferred to 42°C until 48 h of incubation. Cloacae swabs (ducks) were directly inoculated in Preston broth and processed in the same way as fecal samples. After incubation of the enrichment broth, 200 µl were streaked on a Karmali plate and incubated at 42°C for 48 h.

**Suspected *Campylobacter* isolates.** After incubation, each Karmali plate was visually screened for suspected *Campylobacter* isolates. From each plate, one suspected colony was fully identified to species. In the case of morphologically different colonies on the same plate, more that one colony could be identified to species. Species identification was made as described previously .

References for supplemental material and methods

1. Levesque S, St-Pierre K, Frost E, Arbeit RD, Michaud S (2011) Determination of the optimal culture conditions for detecting thermophilic campylobacters in environmental water. J Microbiol Methods 86: 82-88.

2. St-Pierre K, Levesque S, Frost E, Carrier N, Arbeit RD, et al. (2009) Thermotolerant coliforms are not a good surrogate for *Campylobacter* spp. in environmental water. Appl Environ Microbiol 75: 6736-6744.

3. Levesque S, St-Pierre K, Frost E, Arbeit RD, Michaud S (2012) Use of Amplified-Fragment Length Polymorphism to Study the Ecology of *Campylobacter jejuni* in Environmental Water and to Predict Multi-Locus Sequence Typing Clonal Complexes. Appl Environ Microbiol.
