# Supplementary material for: Campylobacteriosis in Urban versus Rural Areas: A Case-Case Study Integrated with Molecular Typing to Validate Risk Factors and to Attribute Sources of Infection
Source: PLoS One. 2013 Dec 26;8(12):e83731. doi: 10.1371/journal.pone.0083731 (PMC3873381; doi:10.1371/journal.pone.0083731)
Supplement: Results S1 — (DOC) [file pone.0083731.s003.doc]

**Supplemental material**

RESULTS

**Regional incidence rates.** The rates varied from 49.2/100,000 in Granit to 211.1/100,000 in Coaticook; these interregional differences were observed for both years of the study (Table 1). The relative risk (RR) represents the incidence rate of campylobacteriosis in one county compared to the incidence rate in the other counties taken as a whole. The risk of campylobacteriosis was 2.93-fold higher in Coaticook (p<0.0001) and 1.62-fold higher in Haut St-François (p=0.02) than elsewhere in the Eastern Townships.

***Clinical presentation of cases.*** Diarrhoea was a presenting complaint in 100% of cases, with a median duration of 7 days (range: 1-180 days). Other presenting findings included abdominal pain (89%), fever (75%), nausea (47%), bloody stools (40%) and vomiting (28%). There was appreciable morbidity; 35% of the cases were hospitalised (median duration: 2 day; range: 1-11 days), and 53% of cases missed work or school (median duration: 4 days; range 1-21 days). One case was hospitalised due to a *C. fetus* bacteremia with septic thrombophlebitis of both legs. There were no deaths.

The median interval from the onset of symptoms until stool culture was 4 days (range: 0-167 days; 90^th^%ile, 9 days), with 5 additional days (range: 1-29 days; 90^th^%ile, 9 days) before notification of the Public Health Department, and 5 more days (range: 0-29 days; 90^th^%ile, 14 days) until the epidemiological investigation. Thus, the median total interval from the onset of symptoms to the interview of the cases was 14 days (range: 6-200 days; 90^th^%ile, 26 days). The majority of intervals over 26 days was explained by the delay between the onset of symptoms and the stool culture.

According to answers to the whole questionnaire, the interviewers tried to determine the probable source of infection. The principal risk factors were chicken (17%), contaminated water (19%), an animal contact (15%), raw milk (16%), other food (10%), an infectious contact (4%), and other sources (2%). In 17%, interviewers could not identify a putative source of infection.
