# Supplementary material for: Campylobacteriosis in Urban versus Rural Areas: A Case-Case Study Integrated with Molecular Typing to Validate Risk Factors and to Attribute Sources of Infection
Source: PLoS One. 2013 Dec 26;8(12):e83731. doi: 10.1371/journal.pone.0083731 (PMC3873381; doi:10.1371/journal.pone.0083731)
Supplement: Table S1 — Incidence rates of campylobacteriosis in each county and for each municipality category in the Eastern Townships (DOC) [file pone.0083731.s004.doc]

Table S1. Incidence rates of campylobacteriosis in each county and for each municipality category in the Eastern Townships ^a^.

|  |  | 2005-2006 (July to June) | | 2006-2007(July to June) | | Total Study^c^ (July 2005 to December 2007) | | | |
| --- | --- | --- | --- | --- | --- | --- | --- | --- | --- |
|  | Total population^b^ | Number of cases | Incidence rate per 100,000 | Number of cases | Incidence rate per 100,000 | Number of cases | Incidence rate per 100,000 | RR | p value |
| Eastern Townships | 298685 | 117 | 39.2 | 78 | 26.1 | 241 | 80.7 | — | — |
| County |  |  |  |  |  |  |  |  |  |
| Asbestos | 14485 | 8 | 55.2 | 4 | 27.6 | 15 | 103.6 | 1.30 | NS |
| Val St-François | 29005 | 12 | 41.4 | 9 | 31.0 | 30 | 103.4 | 1.32 | NS |
| Sherbrooke | 147430 | 40 | 27.1 | 29 | 19.7 | 82 | 55.6 | 0.99 | NS |
| Coaticook | 18475 | 15 | 81.2 | 16 | 86.6 | 39 | 211.1 | 2.93 | < 0.0001 |
| Memphrémagog | 45330 | 18 | 39.7 | 13 | 28.7 | 37 | 81.6 | 1.01 | NS |
| Haut St-François | 21615 | 18 | 83.3 | 4 | 18.5 | 27 | 124.9 | 1.62 | 0.0175 |
| Granit | 22345 | 6 | 26.9 | 3 | 13.4 | 11 | 49.2 | 0.59 | NS |
| Area |  |  |  |  |  |  |  |  |  |
| Rural and Semi-rural | 151255 | 77 | 50.9 | 49 | 32.4 | 159 | 105.1 | 1.89 | < 0.0001 |
| Urban | 147430 | 40 | 27.1 | 29 | 19.7 | 82 | 55.6 | 0.53 |

^a^ Incidence rates were calculated with the cases in the final data set (n = 241).

^b^ Population data is from the 2006 Canadian Census.

^c^ The study period is from 07-2010 to 12-2007. Data from July to December 2007 are shown only in the "Total study".
