# Supplementary material for: Campylobacteriosis in Urban versus Rural Areas: A Case-Case Study Integrated with Molecular Typing to Validate Risk Factors and to Attribute Sources of Infection
Source: PLoS One. 2013 Dec 26;8(12):e83731. doi: 10.1371/journal.pone.0083731 (PMC3873381; doi:10.1371/journal.pone.0083731)
Supplement: Table S2 — Distribution of 851 C. jejuni isolates among clonal complexes, sequence types (ST) and isolation sources. New STs identified in this study and in our previous study (2) are in boldface and new CCs are italicized. (DOC) [file pone.0083731.s005.doc]

Table S2. Distribution of 851 *C. jejuni* isolates among clonal complexes, sequence types (ST) and isolation sources. New STs identified in this study and in our previous study (2) are in boldface and new CCs are italicized.

|  |  | **Source of isolation** | | | | |  |
| --- | --- | --- | --- | --- | --- | --- | --- |
| **CC** | **ST** | **Human** | **Chicken** | **Bovine** | **Water** | **Wild bird** | **Total** |
| ST-21 | 8 | 6 | 6 |  |  | 1 | 13 |
|  | 19 | 2 |  |  |  | 1 | 3 |
|  | 21 | 31 | 10 | 19 | 5 | 1 | 66 |
|  | 50 | 4 | 17 | 1 |  |  | 22 |
|  | 86 |  | 1 |  |  |  | 1 |
|  | 169 | 1 |  |  |  |  | 1 |
|  | 806 | 5 |  | 9 |  |  | 14 |
|  | 982 | 10 |  | 1 |  |  | 11 |
|  | 1209 | 2 |  | 4 | 2 |  | 8 |
|  | 2038 | 1 |  |  |  |  | 1 |
|  | **2862** | 2 | 1 |  |  |  | 3 |
|  | 3857 |  |  | 1 |  |  | 1 |
|  | **3875** | 1 |  |  |  |  | 1 |
|  | **3877** | 1 |  |  |  |  | 1 |
|  | **4018** | 1 |  |  |  |  | 1 |
|  | **4026** |  |  | 1 |  |  | 1 |
|  | **4110** |  | 1 |  |  |  | 1 |
|  | **4377** | 1 |  |  |  |  | 1 |
|  | **4379** |  |  | 1 |  |  | 1 |
|  | **NEW** |  |  |  | 1 |  | 1 |
| **Total ST-21** |  | **68** | **36** | **37** | **8** | **3** | **152** |
| ST-22 | 22 | 1 | 3 |  |  |  | 4 |
| **Total ST-22** |  | **1** | **3** |  |  |  | **4** |
| ST-42 | 42 | 5 | 1 | 5 | 1 | 1 | 13 |
|  | 459 | 4 | 2 | 1 |  |  | 7 |
|  | 2371 |  |  | 1 |  |  | 1 |
|  | **3880** | 1 |  |  |  |  | 1 |
|  | **4109** |  | 1 |  |  |  | 1 |
|  | **4206** |  |  |  | 1 |  | 1 |
| **Total ST-42** |  | **10** | **4** | **7** | **2** | **1** | **24** |
| ST-45 | 45 | 18 | 38 | 1 | 36 | 3 | 96 |
|  | 137 | 3 | 3 |  | 5 |  | 11 |
|  | 168 |  |  |  | 1 |  | 1 |
|  | 241 |  | 1 |  | 3 | 1 | 5 |
|  | 408 |  |  |  |  | 1 | 1 |
|  | 538 | 1 |  |  |  |  | 1 |
|  | 583 |  |  |  |  | 1 | 1 |
|  | 679 | 1 |  |  |  |  | 1 |
|  | 766 |  |  | 1 |  |  | 1 |
|  | 1228 |  | 1 |  |  |  | 1 |
|  | 1363 |  |  |  |  | 1 | 1 |
|  | 2197 |  |  |  | 4 |  | 4 |
|  | 2219 |  |  |  | 3 |  | 3 |
|  | 3730 |  | 1 |  |  |  | 1 |
|  | **3883** |  | 1 |  |  |  | 1 |
|  | **3887** |  | 1 |  |  |  | 1 |
|  | **4024** |  | 1 |  |  |  | 1 |
|  | **4025** |  | 1 |  |  |  | 1 |
|  | **4094** |  |  |  | 1 |  | 1 |
|  | **4114** |  |  |  | 1 |  | 1 |
|  | **4135** |  |  |  | 1 |  | 1 |
|  | **4136** |  |  |  | 1 |  | 1 |
|  | **4191** |  |  |  | 1 |  | 1 |
|  | **4381** |  |  |  | 1 |  | 1 |
| **Total ST-45** |  | **23** | **48** | **2** | **58** | **7** | **138** |
| ST-48 | 38 | 3 |  | 9 |  |  | 12 |
|  | 48 | 1 | 6 |  |  |  | 7 |
|  | 918 | 1 |  | 3 |  |  | 4 |
| **Total ST-48** |  | **5** | **6** | **12** |  |  | **23** |
| ST-49 | 3 |  |  | 1 |  |  | 1 |
|  | **2868** |  | 3 |  |  |  | 3 |
|  | **4020** | 1 |  |  |  |  | 1 |
|  | **4181** |  | 1 |  |  |  | 1 |
| **Total ST-49** |  | **1** | **4** | **1** |  |  | **6** |
| ST-52 | 52 | 7 | 3 |  |  |  | 10 |
|  | 1812 |  | 2 |  |  |  | 2 |
|  | **4127** |  |  |  | 1 |  | 1 |
| **Total ST-52** |  | **7** | **5** |  | **1** |  | **13** |
| ST-61 | 61 | 6 | 2 | 13 | 1 |  | 22 |
|  | 432 |  |  |  | 1 |  | 1 |
|  | 955 | 1 |  |  |  |  | 1 |
|  | 1244 | 1 |  |  |  |  | 1 |
|  | **4017** | 1 |  |  |  |  | 1 |
|  | **4120** |  |  |  | 2 |  | 2 |
|  | **4348** |  |  |  | 1 |  | 1 |
| **Total ST-61** |  | **9** | **2** | **13** | **5** |  | **29** |
| ST-177 | 177 |  |  |  | 3 |  | 3 |
|  | 563 |  |  |  | 1 |  | 1 |
|  | 2539 |  |  |  | 1 |  | 1 |
|  | **4141** |  |  |  | 2 |  | 2 |
|  | **4212** |  |  |  |  | 1 | 1 |
|  | **4213** |  |  |  |  | 1 | 1 |
| **Total ST-177** |  |  |  |  | **7** | **2** | **9** |
| ST-179 | 1207 | 1 |  |  | 2 |  | 3 |
|  | **3889** | 2 |  | 1 | 19 |  | 22 |
|  | **4027** |  |  |  | 1 |  | 1 |
|  | **4031** |  |  |  | 1 |  | 1 |
|  | **4121** |  |  |  | 1 |  | 1 |
|  | **4186** |  |  |  | 2 |  | 2 |
|  | **4189** |  |  |  | 2 |  | 2 |
|  | **4380** |  |  |  | 1 |  | 1 |
| **Total ST-179** |  | **3** |  | **1** | **29** |  | **33** |
| ST-206 | 222 | 3 | 1 | 1 |  |  | 5 |
|  | **4090** | 1 | 2 |  |  |  | 3 |
| **Total ST-206** |  | **4** | **3** | **1** |  |  | **8** |
| ST-257 | 929 | 3 | 1 | 2 |  |  | 6 |
|  | **4022** | 1 |  | 1 |  |  | 2 |
| **Total ST-257** |  | **4** | **1** | **3** |  |  | **8** |
| ST-283 | 267 | 1 | 1 |  | 8 |  | 10 |
|  | **3884** |  | 1 |  |  |  | 1 |
|  | **4180** |  | 4 |  |  |  | 4 |
|  | **4347** |  | 1 |  |  |  | 1 |
| **Total ST-283** |  | **1** | **7** |  | **8** |  | **16** |
| ST-353 | 353 | 1 | 6 |  |  |  | 7 |
|  | 939 |  | 3 |  |  |  | 3 |
|  | 1210 | 1 | 11 |  |  |  | 12 |
|  | 1218 | 1 |  |  |  |  | 1 |
|  | 1898 | 1 |  |  |  |  | 1 |
|  | **3879** | 1 |  |  |  |  | 1 |
|  | **3888** |  | 1 |  |  |  | 1 |
|  | **4019** | 1 |  |  |  |  | 1 |
|  | **4023** |  | 1 |  |  |  | 1 |
|  | **4092** |  | 1 |  |  |  | 1 |
|  | **4182** |  | 5 |  |  |  | 5 |
|  | **4184** |  | 1 |  |  |  | 1 |
| **Total ST-353** |  | **6** | **29** |  |  |  | **35** |
| ST-354 | 354 |  |  |  | 1 |  | 1 |
|  | **3874** | 1 |  |  |  |  | 1 |
|  | **4091** |  | 2 |  |  |  | 2 |
| **Total ST-354** |  | **1** | **2** |  | **1** |  | **4** |
| ST-403 | 933 | 3 |  | 4 |  |  | 7 |
| **Total ST-403** |  | **3** |  | **4** |  |  | **7** |
| ST-443 | 443 |  | 1 |  |  |  | 1 |
| **Total ST-443** |  |  | **1** |  |  |  | **1** |
| ST-460 | 460 | 1 | 2 |  |  |  | 3 |
|  | 535 |  | 7 |  |  |  | 7 |
|  | **3881** |  | 2 |  |  |  | 2 |
|  | **3882** |  | 1 |  |  |  | 1 |
|  | **4099** | 1 |  |  |  |  | 1 |
|  | **4112** |  |  |  | 1 |  | 1 |
| **Total ST-460** |  | **2** | **12** |  | **1** |  | **15** |
| ST-508 | 132 |  |  | 4 |  |  | 4 |
|  | 508 | 1 |  |  |  |  | 1 |
|  | **4111** |  |  | 1 |  |  | 1 |
|  | **4183** |  | 1 |  |  |  | 1 |
|  | **4194** |  |  |  | 1 |  | 1 |
| **Total ST-508** |  | **1** | **1** | **5** | **1** |  | **8** |
| ST-607 | 607 | 1 | 2 |  |  |  | 3 |
|  | 924 |  | 4 |  |  |  | 4 |
|  | 1212 | 4 | 70 |  |  |  | 74 |
|  | **4178** | 1 |  |  |  |  | 1 |
|  | **4179** |  | 1 |  |  |  | 1 |
| **Total ST-607** |  | **6** | **77** |  |  |  | **83** |
| ST-682 | 682 |  |  |  | 5 |  | 5 |
|  | 1027 |  |  |  | 1 |  | 1 |
|  | **4203** |  |  |  | 1 |  | 1 |
| **Total ST-682** |  |  |  |  | **7** |  | **7** |
| ST-692 | 699 |  |  |  | 3 |  | 3 |
|  | **4188** |  |  |  | 1 |  | 1 |
|  | **4378** |  | 1 |  |  |  | 1 |
| **Total ST-692** |  |  | **1** |  | **4** |  | **5** |
| ST-702 | 702 |  | 1 |  |  |  | 1 |
|  | **4132** |  |  |  | 1 |  | 1 |
|  | **4214** |  |  |  |  | 2 | 2 |
| **Total ST-702** |  |  | **1** |  | **1** | **2** | **4** |
| ST-952 | **4028** |  |  |  | 1 |  | 1 |
|  | **4190** |  |  |  | 1 |  | 1 |
|  | **4202** |  |  |  | 1 |  | 1 |
|  | **4208** |  |  |  | 1 |  | 1 |
| **Total ST-952** |  |  |  |  | **4** |  | **4** |
| ST-1034 | 694 |  |  |  | 1 |  | 1 |
|  | 1709 |  | 1 |  |  |  | 1 |
|  | 1956 |  | 1 |  |  |  | 1 |
|  | **3891** |  |  |  |  | 1 | 1 |
|  | **4071** | 1 |  |  | 1 | 1 | 3 |
|  | **4079** |  |  |  |  | 1 | 1 |
|  | **4106** |  |  |  |  | 1 | 1 |
|  | **4113** |  |  |  | 1 |  | 1 |
|  | **4124** |  |  |  | 1 |  | 1 |
|  | **4129** |  |  |  | 1 |  | 1 |
|  | **4133** |  |  |  | 1 |  | 1 |
|  | **4193** |  |  |  | 1 |  | 1 |
|  | **4383** |  |  |  | 1 |  | 1 |
| **Total ST-1034** |  | **1** | **2** |  | **8** | **4** | **15** |
| ST-1150 | **4360** |  | 1 |  |  |  | 1 |
| **Total ST-1150** |  |  | **1** |  |  |  | **1** |
| *ST-1224* | 1224 |  |  |  | 6 |  | 6 |
|  | **4029** |  |  |  | 1 |  | 1 |
|  | **4074** |  |  |  | 1 |  | 1 |
|  | **4076** |  |  |  | 1 |  | 1 |
|  | **4104** |  |  |  | 1 |  | 1 |
|  | **4105** |  |  |  | 1 |  | 1 |
|  | **4130** |  |  |  | 3 |  | 3 |
|  | **4134** |  |  |  | 1 |  | 1 |
|  | **4197** |  |  |  | 1 |  | 1 |
|  | **4352** |  |  |  | 1 |  | 1 |
| **Total *ST-1224*** |  |  |  |  | **17** |  | **17** |
| ST-1275 | 637 |  |  |  | 16 | 14 | 30 |
|  | 1223 |  |  |  |  | 2 | 2 |
|  | 1225 |  |  |  | 1 | 2 | 3 |
|  | 1268 |  |  |  | 2 |  | 2 |
|  | 1275 |  |  |  |  | 1 | 1 |
|  | 1283 |  |  |  | 1 |  | 1 |
|  | 1292 |  |  |  | 4 |  | 4 |
|  | **4021** | 1 |  |  |  |  | 1 |
|  | **4034** |  |  |  |  | 1 | 1 |
|  | **4096** |  |  |  | 1 |  | 1 |
|  | **4101** |  |  |  | 1 |  | 1 |
|  | **4119** |  |  |  | 1 |  | 1 |
|  | **4177** | 1 |  |  |  |  | 1 |
|  | **4200** |  |  |  | 1 |  | 1 |
|  | **4205** |  |  |  | 1 |  | 1 |
|  | **4210** |  |  |  |  | 5 | 5 |
|  | **4215** |  |  |  |  | 1 | 1 |
|  | 4279 |  |  |  | 1 |  | 1 |
|  | **4362** |  |  |  |  | 1 | 1 |
| **Total ST-1275** |  | **2** |  |  | **30** | **27** | **59** |
| ST-1287 | **4032** |  |  |  | 1 |  | 1 |
| **Total ST-1287** |  |  |  |  | **1** |  | **1** |
| ST-1332 | 1276 |  |  |  |  | 1 | 1 |
|  | **3886** |  | 1 |  |  |  | 1 |
|  | **4078** |  |  |  |  | 1 | 1 |
| **Total ST-1332** |  |  | **1** |  |  | **2** | **3** |
| *ST-4102* | 1226 |  |  |  | 3 |  | 3 |
|  | 1230 |  |  |  | 2 |  | 2 |
|  | **2866** | 1 | 1 |  | 2 |  | 4 |
|  | **4102** |  |  |  | 1 |  | 1 |
|  | **4118** |  |  |  | 1 |  | 1 |
| **Total *ST-4102*** |  | **1** | **1** |  | **9** |  | **11** |
| Unassigned | 448 |  |  |  | 1 |  | 1 |
|  | 464 | 1 |  |  |  |  | 1 |
|  | 468 |  |  |  | 1 | 2 | 3 |
|  | 922 | 14 | 2 | 1 |  |  | 17 |
|  | 995 |  | 1 |  |  |  | 1 |
|  | 996 |  |  |  | 1 |  | 1 |
|  | 997 |  |  |  | 1 |  | 1 |
|  | 1030 | 1 |  |  |  |  | 1 |
|  | 1289 |  |  |  | 1 | 2 | 3 |
|  | 1351 |  |  |  | 1 |  | 1 |
|  | 1479 |  |  |  | 3 |  | 3 |
|  | 1748 | 1 |  |  |  |  | 1 |
|  | 1911 |  | 1 |  |  |  | 1 |
|  | 1938 |  |  |  |  | 1 | 1 |
|  | 1959 |  |  |  | 1 |  | 1 |
|  | 1961 |  |  |  | 2 |  | 2 |
|  | 1972 |  | 2 |  |  |  | 2 |
|  | 2090 |  |  |  | 1 |  | 1 |
|  | 2349 |  |  |  | 1 |  | 1 |
|  | 2514 |  |  |  | 2 |  | 2 |
|  | 2875 | 1 |  |  |  | 3 | 4 |
|  | 3644 |  | 1 |  |  |  | 1 |
|  | **3885** |  | 1 |  |  |  | 1 |
|  | **3890** |  |  |  | 1 |  | 1 |
|  | **4030** |  |  |  | 1 |  | 1 |
|  | **4033** |  |  |  | 2 |  | 2 |
|  | **4069** | 1 |  |  |  |  | 1 |
|  | **4073** |  |  |  | 1 |  | 1 |
|  | **4075** |  |  |  | 1 |  | 1 |
|  | **4077** |  |  |  |  | 1 | 1 |
|  | **4080** |  |  |  |  | 1 | 1 |
|  | **4093** |  |  |  | 5 |  | 5 |
|  | **4095** |  |  |  | 1 |  | 1 |
|  | **4097** |  |  |  | 1 |  | 1 |
|  | **4098** |  |  |  | 1 |  | 1 |
|  | **4100** |  |  |  | 1 |  | 1 |
|  | **4103** |  |  |  | 1 |  | 1 |
|  | **4115** |  |  |  | 1 |  | 1 |
|  | **4116** |  |  |  | 1 |  | 1 |
|  | **4117** |  |  |  | 1 |  | 1 |
|  | **4122** |  |  |  | 1 |  | 1 |
|  | **4123** |  |  |  | 1 |  | 1 |
|  | **4125** |  |  |  | 1 |  | 1 |
|  | **4126** |  |  |  | 1 |  | 1 |
|  | **4128** |  |  |  | 1 |  | 1 |
|  | **4131** |  |  |  | 1 |  | 1 |
|  | **4137** |  |  |  | 1 |  | 1 |
|  | **4138** |  |  |  | 1 |  | 1 |
|  | **4139** |  |  |  | 1 |  | 1 |
|  | **4140** |  |  |  | 1 |  | 1 |
|  | **4185** |  | 1 |  |  |  | 1 |
|  | **4187** |  |  |  | 1 |  | 1 |
|  | **4192** |  |  |  | 1 |  | 1 |
|  | **4195** |  |  |  | 1 |  | 1 |
|  | **4196** |  |  |  | 1 |  | 1 |
|  | **4198** |  |  |  | 1 |  | 1 |
|  | **4199** |  |  |  | 1 |  | 1 |
|  | **4201** |  |  |  | 1 |  | 1 |
|  | **4204** |  |  |  | 2 |  | 2 |
|  | **4207** |  |  |  | 1 |  | 1 |
|  | **4209** |  |  |  | 1 |  | 1 |
|  | **4211** |  |  |  |  | 1 | 1 |
|  | **4216** |  |  |  |  | 1 | 1 |
|  | **4217** |  |  |  |  | 1 | 1 |
|  | **4349** |  |  |  | 1 |  | 1 |
|  | **4350** |  |  |  | 1 |  | 1 |
|  | **4351** |  |  |  | 1 |  | 1 |
|  | **4353** |  |  |  |  | 1 | 1 |
|  | **4361** |  |  |  | 1 |  | 1 |
|  | **4364** |  |  |  | 1 |  | 1 |
|  | **4365** |  |  |  | 1 |  | 1 |
|  | **4366** |  |  |  | 1 |  | 1 |
|  | **4367** |  |  |  | 1 |  | 1 |
|  | **4382** |  |  |  |  | 1 | 1 |
| **Total unassigned** |  | **19** | **9** | **1** | **64** | **15** | **108** |
| **Total** |  | **178** | **257** | **87** | **266** | **63** | **851** |
